# Supplementary material for: The interactome of intact mitochondria by cross-linking mass spectrometry provides evidence for coexisting respiratory supercomplexes
Source: Mol Cell Proteomics. 2017 Dec 8;17(2):216–32. doi: 10.1074/mcp.RA117.000470 (PMC5795388; doi:10.1074/mcp.RA117.000470)
Supplement: Supplemental Data [file supp_RA117.000470_133922_0_supp_23978_fzffwf.zip › spectra_annotation/mito_spectra_annotation/110-1-9-1-11-1.pdf]

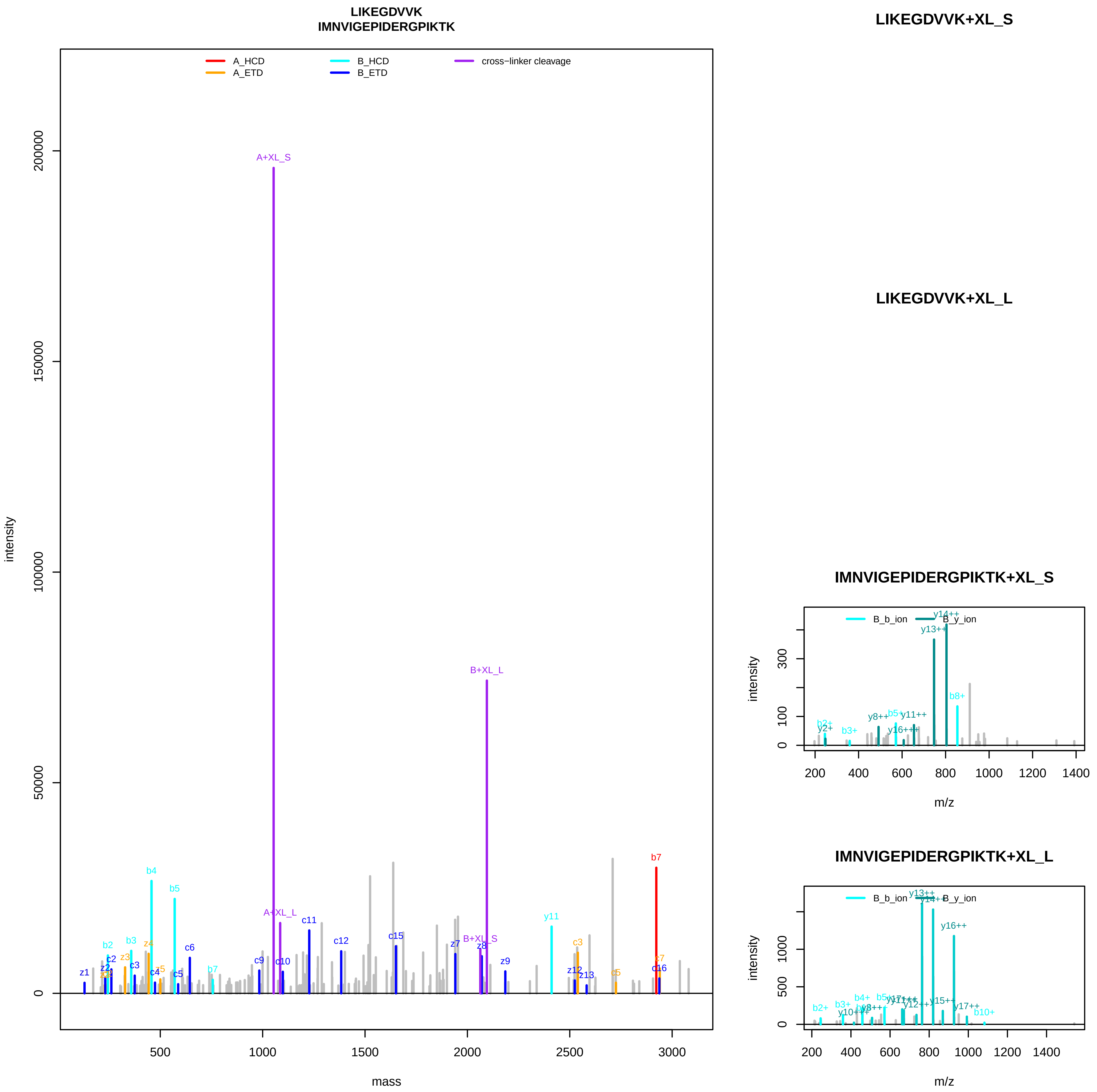

LIKEGDVVK
IMNVIGEPIDERGPIKTK
A_HCD
A_ETD
B_HCD
B_ETD
cross−linker cleavage
intensity
mass
0
50000
100000
150000
200000
500
1000
1500
2000
2500
3000
A+XL_S
B+XL_L
A+XL_L
B+XL_S
b2
b3
b4
b5
b7
y11
b7
z1
z2
c2
z3
c3
z4
c4
z5
c5
c6
c9
c10
c11
c12
c15
z7
z8
z9
c3
z12
z13
c5
c7
c16
LIKEGDVVK+XL_S
LIKEGDVVK+XL_L
IMNVIGEPIDERGPIKTK+XL_S
B_b_ion
B_y_ion
intensity
m/z
0
100
300
200
400
600
800
1000
1200
1400
b2+
y2+
b3+
y8++
b5+
y11++
y16++
y13++
y14++
b8+
IMNVIGEPIDERGPIKTK+XL_L
B_b_ion
B_y_ion
intensity
m/z
0
500
1000
200
400
600
800
1000
1200
1400
b2+
b3+
y10++
b4+
y8++
b5+
y17++
y11++
y12++
y13++
y14++
y15++
y16++
y17++
b10+
